# Xp11.2 translocation renal cell carcinomas in young adults

**DOI:** 10.1186/s12894-015-0055-0

**Published:** 2015-07-01

**Authors:** Linfeng Xu, Rong Yang, Weidong Gan, Xiancheng Chen, Xuefeng Qiu, Kai Fu, Jin Huang, Guancheng Zhu, Hongqian Guo

**Affiliations:** Department of Urology, The Affiliated Drum Tower Hospital of Medical College of Nanjing University, Zhongshan Road 321, Nanjing, Jiangsu Province 210008 China

**Keywords:** Xp11.2 translocation, Renal cell carcinomas, TFE3, FISH

## Abstract

**Background:**

Little is known about the biological behavior of Xp11.2 translocation renal cell carcinomas (RCCs) as few clinical studies have been performed using a large sample size.

**Methods:**

This study included 103 consecutive young adult patients (age ≤ 45 years) with RCC who underwent partial or radical nephrectomy at our institution from 2008 to 2013. Five patients without complete clinical data were excluded. Of the 98 remaining patients, 16 and 82 patients were included in the Xp11.2 translocation and non-Xp11.2 translocation groups, respectively. Clinicopathologic data were collected, including age, gender, tumor size, laterality, symptoms at diagnosis, surgical procedure, pathologic stage, tumor grade, time of recurrence and death.

**Results:**

Xp11.2 translocation RCCs were associated with higher tumor grade and pathologic stage (*P* < 0.05, Fisher’s exact test). During the median follow-up of 36 months (range: 3–71 months), the number of cancer-related deaths was 4 (4.9 %) and 3 (18.7 %) in the non-Xp11.2 translocation and Xp11.2 translocation groups, respectively. The Kaplan-Meier cancer specific survival curves revealed a significant difference between non-Xp11.2 translocation RCCs and Xp11.2 translocation RCCs in young adults (*P* = 0.042).

**Conclusions:**

Compared with non-Xp11.2 translocation RCCs, the Xp11.2 translocation RCCs seemingly showed a higher tumor grade and pathologic stage and have similar recurrence-free survival rates but poorer cancer-specific survival rates in young adults.

## Background

Renal cell carcinoma (RCC) is the most common type of kidney cancer in adults and accounts for approximately 3 % of adult malignancies and 90–95 % of neoplasms arising from the kidney [1]. The morbidity and mortality of RCC is still growing. RCC can be histologically classified into several subtypes, among which clear cell RCC is the most prevalent and represents 70–80 % of kidney cancers [2].

Xp11.2 translocation RCC was first listed as a specific disease entity in the World Health Organization Classification of Tumors in 2004 [3]. This RCC subtype is defined by different translocations involving chromosome Xp11.2, all of which result in transcription factor E3 (TFE3) gene fusions. Several fusions of the TFE3 gene with different genes have been identified to date, including ASPL(17q25), PRCC(1q21), PSF(1q34), NonO(Xq12) and CLTC(17q23) [4]. Another subset of RCC is associated with transcription factor EB (TFEB) resulting from t(6;11)(p21;q12). PRCC-TFE3 RCCs [5] and ASPL-TFE3 RCCs [6] are the most frequent kinds of Xp11.2 translocation RCCs.

Recent reports have shown that the incidence of Xp11.2 translocation RCC is low. Approximately one-third of pediatric RCCs are estimated to be Xp11.2 translocation RCCs associated with TFE3 gene fusion [7]. Several studies have recently evaluated its incidence as 0.9 % (6/632) in adult RCCs [8], 15 % (4/26) in young adult RCCs [9], and 54 % (7/13) in child RCCs [10].

A meta-analysis by Rao et al. [11] demonstrated that TFE3 + pediatric RCCs were associated with a poorer outcome and higher stage (III/IV) than TFE3-RCCs. Komai et al. [12] reported that young patients (≤45 years) with RCC had similar recurrence-free survival rates but better cause-specific survival rates compared with older patients. In that study, Xp11.2 translocation RCCs accounted for at least one half of the young patients with RCC who had developed recurrence.

Until now, few clinical studies have examined the biological behavior of Xp11.2 translocation RCCs in young adults (≤45 years). In this study, we aimed to better define the biological behavior of Xp11.2 translocation RCCs and to determine whether its clinical outcomes differ from those of non-Xp11.2 translocation RCCs in young adults.

We hypothesized that Xp11.2 translocation RCCs have poorer prognosis than non-Xp11.2 translocation RCCs in young adults. The objectives of this study were as follows: (1) to compare the clinicopathologic data of Xp11.2 translocation RCCs with that of non-Xp11.2 translocation RCCs and obtain the clinicopathologic features that correlated with Xp11.2 translocation RCCs, and (2) confirm if cancer-specific survival (CSS) and recurrence-specific survival (RFS) of Xp11.2 translocation RCCs were significantly different from those of non-Xp11.2 translocation RCCs.

## Methods

### Study population

Of the 879 consecutive adult RCCs in our institution from 2008 to 2013, 103 patients were in the age range of 18–45 years. Five cases without complete clinical data were excluded. Of the remaining 98 patients, there were 16 with Xp11.2 translocation RCCs, 61 with clear cell RCCs, 10 with papillary RCCs, 9 with chromophobe RCCs and 2 with unclassified RCCs. In this study, we defined young age as ≤ 45 years according to definitions used in previous studies [9, 12]. We diagnosed Xp11.2 translocation RCCs with positive fluorescence in situ hybridization (FISH) after initial screening according to medical history, age, pathologic morphology and subsequent TFE3 immunostaining. The study was approved by the Committee on Medical Ethics of Nanjing Drum Tower Hospital, Jiangsu, China. All patients provided written informed consent.

### Immunostaining

To investigate the incidence of Xp11.2 translocation RCC, TFE3 immunostaining was performed on paraffin-embedded tissue with the primary antibody TFE3 (Millipore, Billerica MA, US) using the manual overnight incubation methodology (using heat-induced epitope retrieval and the Dako Envision detection system).

### FISH

A dual-color break-apart FISH assay for TFE3 gene rearrangement at the Xp11.2 region was performed on the TFE3 positively stained tissue using a self-designed polyclonal break-apart probe. In brief, FISH of interphase nuclei was performed on 4-μm-thick paraffin-embedded sections. The telomere sides of TFE3 gene cloning fragments (CTD-2516D6, CTD-2522 M13, and RP11-416B14) were labeled with fluorescein-12-dUTP and the centromeric sides of TFE3 gene cloning fragments (CTD-2312C1, CTD-2248C21, and RP11-959H17) were labeled with tetramethylrhodamine-5-dUTP. After sample preparation, hybridization with labeled DNA was performed overnight. Slides were counterstained with 4, 6-diamidino-2-phenylindole (DAPI, Vysis, Abbott Park, IL, USA) and analyzed using an Olympus BX-51 fluorescence microscope (Center Valley, PA, USA). Co-localization of red and green signals in tumor nuclei was considered negative, and a split signal in more than 10 % tumor nuclei was regarded positive for TFE3 rearrangement.

### Assessment

The collected clinicopathologic data were as follows: age, gender, tumor size, laterality, symptoms at diagnosis, surgical procedure, pathologic stage, and tumor grade. All patients presented with tumor-free status after nephrectomy because no surgery was conservative or cytoreductive.

All patients had undergone a thorough medical history interview, physical examination, radiographic staging according to the computed tomography and/or magnetic resonance imaging of the abdomen as well as chest radiography. If warranted by the patient symptoms or physical examination findings, bone scans and brain imaging were performed.

The characteristics of the 98 patients are summarized in Table 1. The patients were followed up every 3–12 months with imaging studies. At each consultation, the patient’s status (alive or dead) and the degree of tumor progression were determined. In the present study, the endpoints of follow-up were CSS and RFS.

**Table 1:** Patient and tumor characteristics

Variable	non-Xp11.2 translocation group (*n* = 82)	Xp11.2 translocation group (*n* = 16)	*P* value
Age(y)			0.296
Media(Range)	40 (18–45)	27 (21–40)	
Gender(n)			0.086
Male	56 (68.3 %)	7 (43.8 %)	
Female	25 (31.7 %)	9 (56.3 %)	
Size(cm)			0.588
Media(Range)	4.6 (1.8–17.0)	4.5 (3.0–11.5)	
Laterality(n)			1.000
Left	39 (47.6 %)	7 (43.8 %)	
Right	43 (52.4 %)	9 (56.3 %)	
Symptoms(n)			
Asymptomatic	57 (69.5 %)	11 (68.8 %)	
Symptomatic	25 (30.5 %)	5 (31.3 %)	1.000
Nephrectomy (n)			0.156
Radical	49 (59.8 %)	13 (81.3 %)	
Partial	33 (40.2 %)	3 (18.8 %)	
Overall stage(n)			0.026
I	57 (69.5 %)	10 (62.5 %)	
II	22 (26.8 %)	2 (12.5 %)	
III	2 (2.4 %)	3 (18.8 %)	
IV	1 (1.2 %)	1 (6.3 %)	
TNM(2010AJCC)			
T(n)			0.026
T1	57 (69.5 %)	10 (62.5 %)	
T2	22 (26.8 %)	2 (12.5 %)	
T3	2 (2.4 %)	3 (18.8 %)	
T4	1 (1.2 %)	1 (6.3 %)	
N(n)			0.013
N_0_	81 (98.8 %)	13 (81.3 %)	
N_1_	1 (1.2 %)	3 (18.8 %)	
M(n)			
M_0_	82 (100 %)	16 (100 %)	
M_1_	0 (0)	0 (0)	
Tumor grade (n)			0.011
Low	25 (30.5 %)	0 (0 %)	
Medium	38 (46.3 %)	9 (56.3 %)	
High	19 (23.2 %)	7 (43.8 %)	
Follow-up (mo)			0.464
Media(Range)	33.5 (7–71)	29 (3–70)	

### Statistical analysis

The intergroup differences in the categorical and continuous variables were analyzed using Fisher’s exact test and Student’s *t* test, respectively. The CSS and RFS curves were obtained for Xp11.2 translocation and non-Xp11.2 translocation groups using the Kaplan-Meier method and compared using a log-rank test. All statistical analyses were performed using SPSS, version 17. In all analyses, calculated *P* values of < 0.05 were considered to indicate significance.

## Results

Patients’ outcome and pathologic results are shown in Table 1. The Xp11.2 translocation RCCs were significantly associated with higher tumor grade and pathologic stage (*P* < 0.05, Fisher’s exact test). No statistically significant difference was observed in age, gender, tumor size, laterality, symptoms at diagnosis, or surgical procedure.

**Fig. 1:**
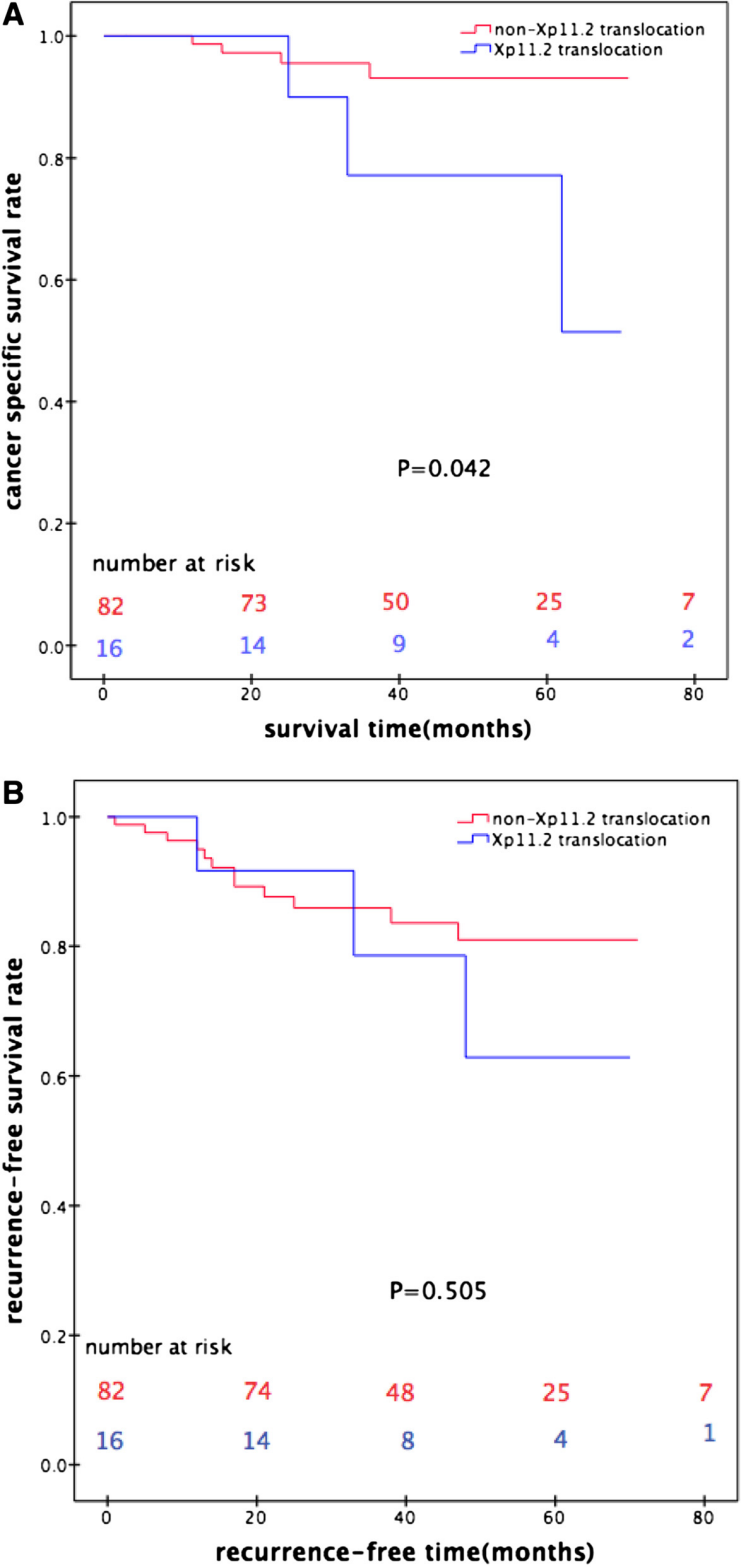
Cancer-specific survival (**a**) and recurrence-specific survival (**b**) analyses were computed comparing non-Xp11.2 translocation renal cell carcinomas (RCCs) with Xp11.2 translocation RCCs in young adults. Red line: non-Xp11.2 translocation RCC; blue line: Xp11.2 translocation RCC

The number of cancer-related deaths was 4 (4.9 %) and 3 (18.7 %) in the non-Xp11.2 translocation and Xp11.2 translocation groups, respectively. Analyses of CSS curves indicated that Xp11.2 translocation RCCs were significantly more frequently associated with a poorer outcome than non-Xp11.2 translocation RCCs (*P* = 0.042, Fig. 1a).

A total of 12 (14.6 %) and 3 patients (18.7 %) in non-Xp11.2 translocation and Xp11.2 translocation groups developed recurrence, respectively. The Kaplan-Meier RFS curves revealed no difference between these two groups (*P* = 0.505, Fig. 1b).

## Discussion

Xp11.2 translocation RCC has been recognized as a distinct entity in the World Health Organization renal tumor classification scheme for 11 years. Its diagnosis is usually based on microscopic appearance and TFE3 immunostaining. Further diagnostic testing is difficult because fresh tissue collection for cytogenetics and molecular analysis is not routinely performed in adult RCCs. Polymerase chain reaction can also be used to confirm a specific gene translocation on formalin-fixed, paraffin-embedded tissue, but it is infrequently used as a clinical diagnostic tool and is more often used in the research setting. At present, the TFE3 break-apart FISH assay has been used to further confirm diagnosis of Xp11.2 translocation RCC [13–16].

The incidence of Xp11.2 translocation RCC is low. Previous studies have revealed an incidence of 0.9 (6/632) [8] to 5 %(6/121) [17] in all adult RCCs and 15 % (4/26) in young adult RCCs [9]. According to age at the time of surgery, the incidence values of TFE3 positivity in the age ranges of 0–10, 11–20, 21–30, and 31–40 years were 67 (2/3), 75 (3/4), 29 (2/7), and 14 % (6/44), respectively (*P* < 0.001) [18]. Because RCC is more commonly encountered in the adult population, the amount of Xp11.2 translocation RCCs in adults may exceed that in the pediatric group. Our study revealed an incidence of 1.8 % (16/879) in all adult RCCs and 15.5 % (16/103) in young adult RCCs, which was consistent with previous reports.

Currently little is known concerning the biological behavior of Xp11.2 translocation RCCs because few clinical studies have been performed with a large sample size.

Based on the available data, the pediatric Xp11.2 translocation RCC is relatively inert, and its prognosis is better than that of adult Xp11.2 translocation RCC [19, 20]. Song et al. [21] reported that pediatric Xp11.2 translocation RCC easily invaded regional lymph nodes and was highly malignant. However, patients with N + M0 maintained a favorable prognosis following surgery alone.

Xp11.2 translocation RCCs that occur in adults may be more aggressive than those in children. Argani et al. [22] investigated 28 adult patients with Xp11.2 translocation RCC, including 16 patients with stage III–IV cancers. Lymph node metastasis occurred in 11 of 13 patients who could be evaluated. Meyer [23] examined 5 adult patients with Xp11.2 translocation RCC, all of whom were in the late stage of their disease with distant metastasis, rapid disease course, and poor outcomes with an average survival of 18 months. Of the 7 adult patients with Xp11.2 translocation RCC that Komai et al. [9] investigated, 5 were classified as stages III–IV and 2 died within 1 year. In a study by Zou et al. [24], the authors reported that 5 out of 9 Xp11.2 RCC patients presented with TNM stages 3–4, and 6 died 10 months to 7 years after their operation. According to the review by Armah and Parwani [20], clinical and pathological heterogeneity may exist between pediatric Xp11.2 translocation RCC and adult Xp11.2 translocation RCC. Xp11.2 translocation RCCs had a high degree of invasiveness, rapid disease course, and poor prognosis in adolescents and adults over the age of 16 years, compared to that in children.

Xp11.2 translocation RCCs were extremely uncommon after 45 years of age, but this is likely an underestimation. Four patients reported by Arnoux [25] were older than 45 years, including three women (53, 71, and 75 years old) and one man (86 years old). One patient was metastatic at diagnosis. Radical nephrectomy was first performed in all cases. TNM staging was T3aN2R0, T3bN0R0, T2N2R0, and T3aN2R2, with a Furhman grade of 4. Two patients progressed with metastasis 5 and 7 months after surgery, and two with lymphatic invasion 2 and 9 months after nephrectomy. One patient died during follow-up. Ellis et al. [26] confirmed that older age or advanced stage at presentation predicted death through multivariate analysis.

In this study, among 16 young adults with Xp11.2 translocation RCCs, 4 (25 %) were classified stage III–IV and 7 (43.8 %) were Furman’s grade 3–4. The Kaplan-Meier CSS curve revealed a significant difference between non-Xp11.2 translocation and Xp11.2 translocation groups. The results of the present study indicated that Xp11.2 translocation RCCs are associated with higher tumor grade and pathologic stage and poorer CCS in young adults.

Based on morphological appearance, RCC is subdivided into clear cell (70–80 %), papillary (10–15 %), chromophobe (3–5 %), collecting duct (1 %), and unclassified (1 %) subtypes [27]. Several studies have shown age to influence the distribution of histological subtypes [12, 28]. A more consistent finding across several studies is that the proportion of tumors with chromophobe histology decreases with increasing age [12, 29, 30]. The clinical behavior of chromophobe RCCs is less aggressive than that of clear cell RCCs, independent of Fuhrman grade or tumor size [31]. The change of histological subtypes may be associated with better prognosis of non-Xp11.2 translocation RCCs.

Similar to conventional RCCs, radical nephrectomy is recommended for Xp11.2 translocation RCCs. Nephron-sparing surgery is an alternative with favorable outcomes in symptomless small RCCs [32]. For the treatment of adult metastatic Xp11.2 translocation RCCs, VEGF-targeted agents appear to demonstrate some efficacy [33].

Our study had several limitations. The sample size was small due to low incidence of this rare disease and the follow-up time was relatively short. The calculation was weak to answer the hypothesis. Thus, we should interpret the CSS curve with some caution before further follow-up is performed.

## Conclusions

Our study showed that Xp11.2 translocation RCCs were seemingly associated with higher tumor grade and pathologic stage in young adults. Moreover, it seemed that Xp11.2 translocation RCCs had similar RFS rates but poorer CSS rates than non-Xp11.2 translocation RCCs in young adults. Our findings suggest that Xp11.2 translocation RCCs should be treated more actively and monitored by follow up.

## Abbreviations

RCC: Renal cell carcinoma
TFE3: Transcription factor E3
TFEB: Transcription factor EB
FISH: Fluorescence in situ hybridization
CSS: Cancer specific survival
RFS: Recurrence-free survival
